# The effect of increasing the illumination on operators’ visual performance in the control-room of a combined cycle power plant

**DOI:** 10.1186/s40557-018-0267-3

**Published:** 2018-08-29

**Authors:** Seyyed Abolfazl Zakerian, Saeid Yazdanirad, Seifollah Gharib, Kamal Azam, Asma Zare

**Affiliations:** 1 Department of Occupational Health, School of Health, Tehran University of Medical Sciences, Tehran, Iran; 2 Department of epidemiology and biostatistics, School of public health, Tehran University of medical sciences, Tehran, Iran; 3 Student Research Committee, Department of Occupational Health, School of Health, Shiraz University of Medical Sciences, Shiraz, Iran

**Keywords:** Lighting, Control room, Visual performance, Visual acuity, Power plant

## Abstract

**Background:**

Lighting is one of the environmental factors affecting the performance of the control room operators. Therefore, the aim of this study was to compare the control room operators’ visual performance in two different illumination conditions at a combined cycle power plant through field-work.

**Methods:**

Sixteen operators in day shift were evaluated with the Freiburg Visual Acuity test (FrACT) software at two lighting systems with different intensities. It includes the first phase with fluorescent illumination system (Power: 40 W, Color Temperature: 4000 Kelvin, Luminous Flux: 2780 Lumen and Model: Pars Shahab) and the second phase with a combined illumination system includes fluorescent and LED (Power: 48 W, Color Temperature: 4000 Kelvin, Luminous Flux: 5400 Lumen and Model: Mazinoor).

**Results:**

Based on the results, visual performance index and visual acuity significantly increased after the intervention (*p* < 0.001). As to contrast, more lighting significantly reduced the percentage of recognized contrast (*p* < 0.001) and increased the contrast performance index (*p* < 0.001).

**Conclusions:**

The results of this study showed that increasing the intensity of light from the values below the allowable limit to the values above the allowable limit would increase the visual indicators in individuals.

## Background

Human direct participation in the production process has diminished by human’s increasing progress in various fields, and instead, the role of the human factor in directing work systems through the control room has increased [1]. In these kinds of tasks, the control of a large difference and sometimes critical parts are taken by human element, and if users cannot process the data quickly and accurately, an error may occur which leads to the occurrence of great events [2]. Human error has long been considered as one of the most important factors in the occurrence of accidents. According to various studies, human errors in complex industries and systems, such as chemical processes, power plants and nuclear power plants, are known as the main causes of accidents. McCafferty (1995) stated that about 80% of the incidents include human error [3]. In Lowe’s study (2004), it was found that 64% of the accidents were because of human error. Other studies conducted by Gatchpole et al.(2006) and Krikos and Baker (2007) also showed that human error was involved in most of the events of complex system [4]. Stringfellow (2010) also showed that 30 to 100% of industrial accidents were caused by human factor [5].

There are various environmental factors which can affect the operators’ performance in the control-room and lead to errors. Conditions of work environment such as heat, sound and illumination have an important effect on people’s attitudes, behavior and performance [6]. Illumination is one of the most important factors in performance. Because most of the activities in processing the data are done by human’s vision system. Therefore, visual performance is very important for control-room operators. The results of the studies show that proper illumination has a positive effect on individual’s performance and reduces the occurrence of accidents [7–10]. Also, it was found in studies that inappropriate illumination increases the eye fatigue, decreases the performance, and eventually leads to an incident. If environmental conditions, especially illumination, cannot meet the individual’s performance requirements, it will reduce the quality of performance and lead to the occurrence of errors. Thus, creating good illumination is very important to individual’s proper performance [9]. Various illumination factors (such as intensity and color temperature) affect the individuals’ visual and cognitive performance [11]. Various factors such as color contrast, illumination level, viewing time, individual differences, gazing and etc., are effective on visual performance [12]. The individual’s efficiency in the control-room is directly affected by visual performance [10]. People need good illumination in the control-rooms to have an appropriate vision [13]. Most studies have investigated the effect of illumination on the individuals’ visual performance in laboratories, so the aim of this study was to compare the control room operators’ visual performance in two different illumination conditions at a combined cycle power plant through field-work.

## Methods

### Participants, time and place of the study

This interventional study was carried out in the summer of 2017. The subjects were the male staff of the control-room in “Parand Power Plant of MAPNA co.” including 16 operators in 12-h shifts (from 7 am to 7 pm and from 7 pm to 7 am) in groups of 4 persons. From the 12-h shift, individuals spent 11 h on visual inspection in the control room. The control-room of the Parand power plant was 10.5 m * 25.5 m and 3.5 m high. There were 4 desks in this control-room where each of them were workstations for all the staff. The desk on which the staff focused was selected as the evaluation desk for intervention. There were 100 fluorescent lamps with white light (Power: 40 W, Color Temperature: 4000 Kelvin, Luminous Flux: 2780 Lumen and Model: Pars Shahab) which were installed in the control-room. They were installed in the ceiling, 50 double-lamps in 10 rows and in each there were 5. The walls were cream color and had a reflection coefficient of 0.7. The floor was gray with a reflection coefficient of 0.5. The roof was matte gray with a reflection coefficient of 0.3. The control-room had 5 windows:two south windows, two western windows and one north window.

### Tools and method of collecting information

The Lux Meter (HAGNER S3) with a precision of 0.01 was used to measure the general illumination. General illumination of the control-room was measured by the network method. The average illumination intensity of the control-room was measured by Lux Meter at a height of 1.2 m based on the fourth model of the Illumination Engineers Society of North America (IESNA). Hagner S3 which is a combined machine to measure the illumination and luminance was used to measure local illumination and luminance.

The photocell of the Lux Meter was placed on the table horizontally, and the level of local luminance was measured in front of each display on the work surfaces. There were two displays on each table in the control room. The illumination was measured in front of both displays at work surface. The average local illumination of the work surface for each display was calculated based on the average of three levels of measurement in front of it.Then, the luminance level of the work surface and the display was measured by the Hagner apparatus.

The Freiburg Visual Acuity test (FrACT) software was used to evaluate the individuals’ visual performance. This software was presented by Michael Bach in 1996 to measure the visual acuity [14]. The validity of this software has been proven in the previous study of visual performance [15]. Two tests of this software -Acuity C and Contrast C- was used to evaluate the visual performance.

The background color is white and the C color is black in the Acuity C test. The direction and size of the letter C is changed in each trial. In order to determine the level of visual performance in this test, the performance indicator will be calculated by Eq. 1:1

Where n is the total correct answers in each trial and τ is the duration of total trials in seconds (The individuals’ reaction time is determined for each 18 trials separately).

In addition to calculating the visual performance indicator, this software also shows visual acuity for each Acuity C test ($$ \raisebox{1ex}{$1$}\!\left/ \!\raisebox{-1ex}{$ arcmin$}\right. $$). It indicated that the higher the value, the greater the individual’s ability to recognize smaller dimensions. Dimension means the opening mouth of the C is in the test.

**Fig. 1:**
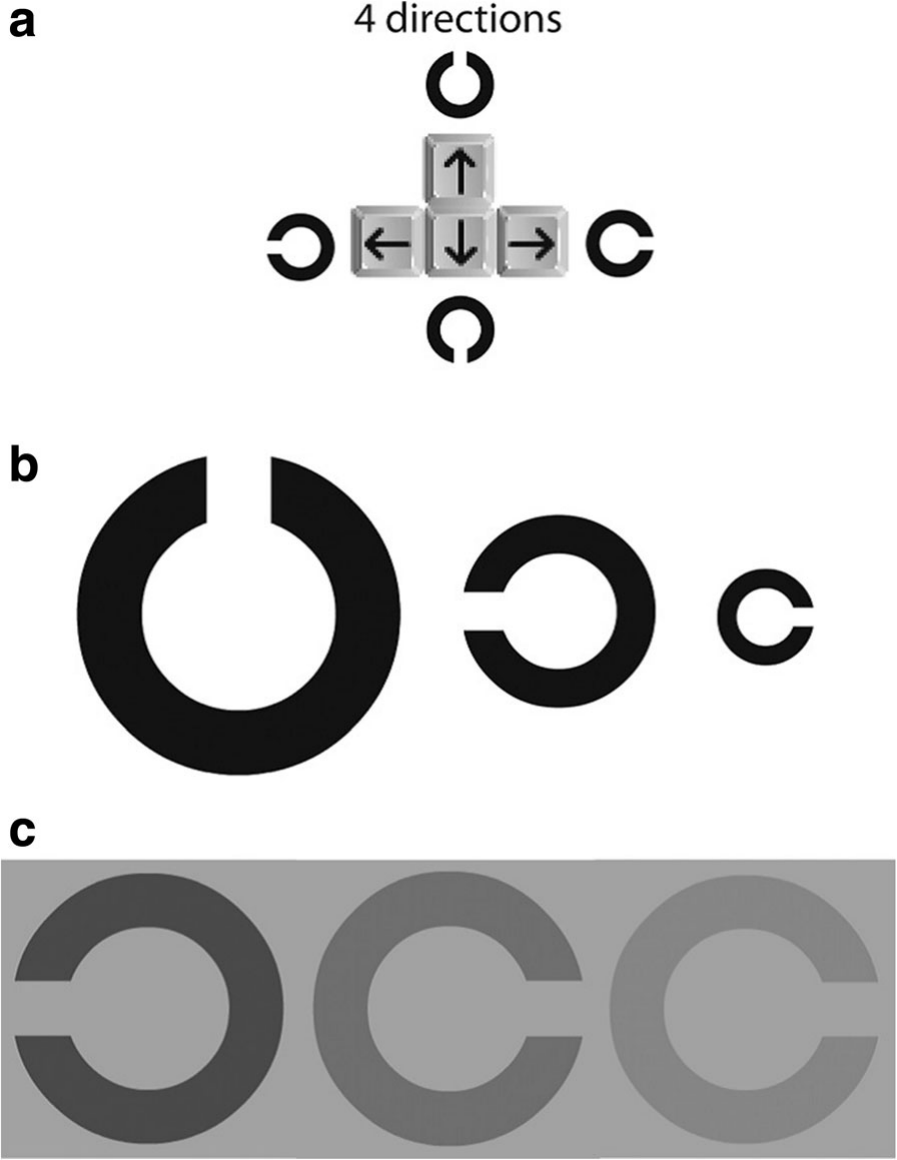
The example of the four main directions of the responses (**a**), aciuty C test (**b**), contrast C test (**c**)

In addition to changing the direction, also the background contrast and the letter C will be changed in the Contrast C test. The subject is asked to use chance whenever he did not see the variable. The performance indicator is also calculated based on the total correct responses in the total duration (second) of the trials in this test. In addition, the results of the Contrast C test are expressed in terms of the percentage of contrast (% contrast Weber). The more a person performs the test carefully, the less the obtained number will be. It indicates that the subject detected the smallest contrast. Response time for each trial is 30 s in the software. If no response is received within this time, the next trial will begin and this non-response will be calculated as the wrong answer. In Fig. 1, an example of the four main directions of the responses (a), acuity C test (b), contrast C test (c) was shown [16].

### The phases of the study

**Fig. 2:**
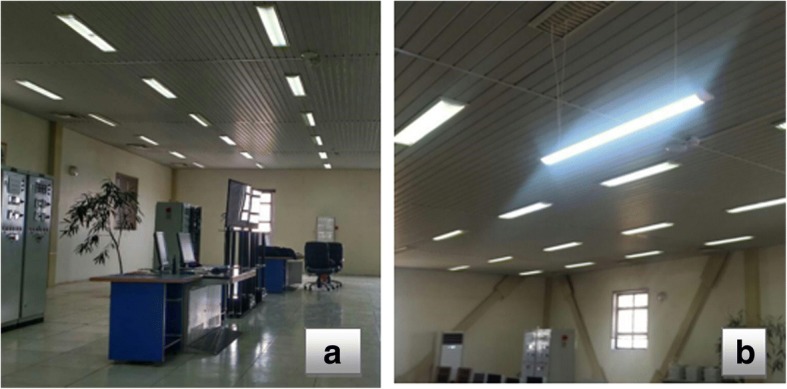
View of the room before and after the intervention (**a**. before the intervention, **b**. after the intervention)

This study was conducted in two phases with interference. It includes the first phase with fluorescent illumination system and the second phase with a combined illumination system includes fluorescent and Light Emission Diodes (LED). The first phase illumination included fluorescent lamps (Power: 40 W, Color Temperature: 4000 Kelvin, Luminous Flux: 2780 Lumen and Model: Pars Shahab), to which people have been exposed for many years. In the first phase, the intensity of general and local illumination, and luminance were measured in two steps including day shift (9:00 and 15:00) and night shift (21:00 and 3:00) and it was in the beginning and the end of each shift. Then individual’s visual performance was evaluated at four times of the day - including morning (at 9:00), afternoon (at 15:00), night (at 21:00), and morning (at 3:00) before and after the intervention. In the next phase, the illumination system was changed and LED lamps (Power: 48 W, Color Temperature: 4000 Kelvin, Luminous Flux: 5400 Lumen and Model: Mazinoor) with the same color temperature with fluorescent lamps were added to the system over the desk to increase the illumination of 200 lx at the work surface.The related calculations about local illumination design were used to calculate the height and suitable number of lamps. Then, a light bulb including 2 LED lamps at a height of 1.5 m above the desk (1 m below the ceiling) was used. In the second phase, for adapting the staff to the new illumination conditions, 15 days were considered. After placing the new illumination system above the desk in the second phase –and after 15 days interval between the evaluations, the intensity of local illumination was measured on this table. Then, the evaluations of the first phase were repeated and recorded (Fig. 2).

### Data analysis

Statistical analysis was performed using SPSS 22.0 software. Wilcoxon non-parametric statistical test was used to compare the two illumination conditions because the data did not follow normal dispersion.

## Results

The mean (Standard Deviation) of age and work experience of the subjects was 36.68 (2.982) years and 10.55 (2.191) years, respectively. The average values of the general and local illumination and the values of day and night luminance were measured before and after the intervention. They are shown in Table 1. The results showed that the illumination level of daylight and night at both work surfaces was less than 300 lx before the intervention (i.e. the suitable lighting for office work according to the standard). A standard luminance to detect the right color is at least 3 Cd / m^2^. The measurement results showed that the displays luminance and working surfaces was suitable for color recognition in daylight -even before doing the study-, while this amount was below the standard level at night.

**Table 1:** Average values of general and local illumination and luminance before and after the intervention

Parameters		Average of general illumination in control-room (Lux)	Illumination of working surface1 (Lux)	Luminance of display1 (Cd/m^2^)	Luminance of working surface1 (Cd/m^2^)	Illumination of working surface2 (Lux)	Luminance of display2 (Cd/m^2^)	Luminance of working surface2 (Cd/m^2^)
Before intervention (first phase)	Day	302	230	6.3	4.2	240	7.1	5.4
	Night	180	153	0.16	0.12	135	0.05	0.03
After intervention (second Phase)	Day	302	415	15.2	12.9	426	16.2	14.8
	Night	180	350	0.2	0.18	332	0.13	0.1

Values of visual performance indicator and visual acuity of Acuity C Test are shown in Table 2 at four times of the day - including morning (at 9:00), afternoon (at 15:00), night (at 21:00), and morning (at 3:00) before and after the intervention.The results of the Wilcoxon test showed that the change of illumination through intervention caused significant changes in performance indicator and visual acuity.

**Table 2:** The values of visual performance indicator of Acuity C Test (Ŋ- accuracy C) and visual acuity before and after the intervention

Time		Before intervention (Median ± IQR)	After intervention (Median ± IQR)	*p*-value^*^
Parameters				
9:00 *N* = 16	Ŋ	0.33 ± 0.09	0.5 ± 0.12	< 0.001
	Visual acuracy ($\frac{1}{Arcmin}$)	1.62 ± 0.34	1.84 ± 0.38	< 0.001
15:00 *N* = 16	Ŋ	0.22 ± 0.12	0.32 ± 0.11	< 0.001
	Visual acuracy ($\frac{1}{Arcmin}$)	1.31 ± 0.56	1.52 ± 0.66	< 0.001
21:00 *N* = 16	Ŋ	0.33 ± 0.12	0.59 ± 0.1	< 0.001
	Visual acuracy ($\frac{1}{Arcmin}$)	1.20 ± 0.41	1.51 ± 0.31	< 0.001
3:00 *N* = 16	Ŋ	0.25 ± 0.13	0.34 ± 0.13	< 0.001
	Visual acuracy ($\frac{1}{Arcmin}$)	1.02 ± 0.24	1.23 ± 0.23	< 0.001

*Wilcoxon Signed Ranks test

The values of visual performance indicator and visual acuity of the contrast C test are shown in Table 3 at four times of the day including morning (9 o’clock), afternoon (at 15 o’clock), night (o’clock 21), and morning (at 3 o’clock) before and after the intervention. The results of the Wilcoxon test showed that the change in illumination through intervention caused significant changes in the values of the performance indicator and the percentage of the contrast.

**Table 3:** The values of performance indicator of Contrast C test (Ŋ- contrast C) and percentage of contrast before and after the intervention

Time		Before intervention (Median ± IQR)	After intervention (Median ± IQR)	*p*-value^*^
Parameters				
9:00 *N* = 16	Ŋ	0.2 ± 0.12	0.41 ± 0.12	< 0.001
	Percentage of Contrast (%)	0.75 ± 0.26	0.52 ± 0.25	< 0.001
15:00 *N* = 16	Ŋ	0.14 ± 0.08	0.31 ± 0.09	< 0.001
	Percentage of Contrast (%)	1.51 ± 0.9	1.39 ± 0.88	< 0.001
21:00 *N* = 16	Ŋ	0.22 ± 0.08	0.53 ± 0.08	< 0.001
	Percentage of Contrast (%)	0.55 ± 0.32	0.43 ± 0.31	< 0.001
3:00 *N* = 16	Ŋ	0.11 ± 0.06	0.31 ± 0.07	< 0.001
	Percentage of Contrast (%)	1.13 ± 0.64	1.04 ± 0.64	< 0.001

*Wilcoxon Signed Ranks test

The results of comparing the values of visual performance indicators at four different times of the day before and after the intervention are shown in Table 4. The results of the statistical test showed that the differences in the values of visual performance indicators at different times of the day were significant (*p* < 0.001).

**Table 4:** Comparison of the values of visual performance indicators before and after the intervention

Before Intervention	9:00	15:00	*p*-value	21:00	3:00	*p*-value
Ŋ-acuity C *N* = 16	0.33 ± 0.09	0.22 ± 0.12	< 0.001^*^	0.33 ± 0.12	0.25 ± 0.13	< 0.001^*^
Visual acuracy ($\frac{1}{Arcmin}$) *N* = 16	1.62 ± 0.34	1.31 ± 0.56	< 0.001^*^	1.20 ± 0.41	1.02 ± 0.24	< 0.001^*^
Ŋ-contrast C *N* = 16	0.2 ± 0.12	0.14 ± 0.08	< 0.001^*^	0.22 ± 0.08	0.11 ± 0.06	< 0.001^*^
Percentage of Contrast (%) *N* = 16	0.75 ± 0.86	1.51 ± 0.9	< 0.001^*^	0.55 ± 0.32	1.13 ± 0.64	< 0.001^*^
After Intervention	9:00	15:00	*p*-value	21:00	3:00	*p*-value
Ŋ-acuity C *N* = 16	0.5 ± 0.12	0.32 ± 0.11	< 0.001^*^	0.59 ± 0.1	0.34 ± 0.13	< 0.001^*^
Visual acuracy ($\frac{1}{Arcmin}$) *N* = 16	1.84 ± 0.38	1.52 ± 0.66	< 0.001^*^	1.51 ± 0.31	1.23 ± 0.23	< 0.001^*^
Ŋ-contrast C *N* = 16	0.41 ± 0.12	0.31 ± 0.09	< 0.001^*^	0.53 ± 0.08	0.31 ± 0.07	< 0.001^*^
Percentage of Contrast (%) *N* = 16	0.52 ± 0.85	1.39 ± 0.88	< 0.001^*^	0.43 ± 0.31	1.04 ± 0.64	< 0.001^*^

*Wilcoxon Signed Ranks test

## Discussion

The results of the present study indicated that the lighting system with intensity of 200 lx resulted in the reduction of the visual performance. On the other hand, the lighting system with intensity of 400 Lux improved visual performance. Therefore, increasing the illumination for administrative work - from the values which are below the permitted level to a higher level- would increase the visual indicators, visual accuracy, and the percentage of the contrast in individuals. Indeed, the increased mean value of the illumination created a actual improvement in the visual performance of the actual working condition. According to the results shown in Table 1, the values of day and night local illumination at working surfaces increased to a level higher than 300 lx after installation of LED lamps. Unfortunately, the luminance level of the surfaces was less than the permitted level at night -even after the intervention- and it requires a lamp with a higher color temperature. The luminance of the display surface was affected by illumination [17–19] and the high intensity of the illumination faded the display images in the user’s eyes [20], so we chose an illumination of about 400 lx for the intervention in illumination. On the other hand, there was no significance difference between the luminance stemmed from the light reflection in the average illumination and the light reflection in the low illumination (about 200 lx). By increasing the illumination from 200 lx to 400 lx, no change in the luminance will occur [21]. However, the results of statistical tests in Tables 2 and 3 show the effect of illumination on individuals’ response rate to visual trials. These results are in line with the previous studies, which showed that illumination has a completely direct effect on visual performance [17–19]. According to previous studies, the illumination of 200 lx causes eye fatigue [22]. Lin in his study concluded that the illumination of about 500 lx provided a better visual performance than 200 lx and 1000 lx [22]. Generally, the results of the present study showed that the visual performance in illumination of 400 lx was much better than that of 200 lx. According to these results, it can be argued that the illumination of 400 lx will improve the eye performance without visual discomfort. These results are in line with those of a study by Shieh et al. in 2000. They investigated the effects of illumination and type of the display on the visual performance of computer users and stated that the visual performance in illumination of 450 lx was greatly improved compared to 200 lx, and computer users experienced more visual comfort at the illumination of 450 lx [23]. Some previous field studies were also consistent with the present study. Juslen et al. investigated the effect of illumination changes on the staffs’ visual performance in a food industry. They concluded that increasing the local illumination by adding additional lamps to the general illumination system over the workstations would improve the visual performance and increase the staff’s satisfaction in the production line at the workplace [24]. In general, the results of the previous studies show that reducing visual fatigue and improving visual performance, reduce the error rate and improve the quality of the work performance [9]. Therefore, it can be concluded that increasing the intensity of light can reduce the effects of eye fatigue such as headache and eye pain and can also prevent occupational accidents by improving work quality.

The effect of illumination on the visual performance has been studied in the past [25], but previous studies are not perfect because they did not study the effect of white light in a real environment on individuals and also did not examine the visual performances such as visual acuity. Light improves the visual performance through visual systems. In a study by Lin et al., it was shown that light improved the performances associated with the visual system through the visual system [25]. In addition to the above mentioned points, the effect of two types of lamps with the same color temperature was examined on visual performance in this study. At the same color temperature (4000 Kelvin), the combined LED lighting and fluorescent lighting system improves visual performance and reduces the individuals’ reaction time in performing visual tasks. These results are in line with the study of Linhart, which states that using LED lighting systems in industrial environments can have positive effects on visual performance and reduced eye fatigue [16]. The LED system supports optimal visual performance more than the fluorescent illumination system with a fairly low color temperature. The LED-based illumination system does not create gazing, so it leads to more support of optimal visual performance [26].

The results also showed that the shift time had a significant effect on individuals’ working memory performance and caused a decrease in the response speed at the end of the day shift. According to the results, time affects the response time and the number of correct answers in the acuity C duty and contrast C duty, so that response time and visual error increased at the end of the shift compared to the beginning of the shift, and the number of correct answers in both duties decreased. In other words, two visual performances at the end of day shifts decreased significantly compared to the beginning of the shift and also suffered a decline. These variables have not been studied in a field studies, but generally, the results of this study are consistent with those of previous studies about visual performance [27]. Factors influencing visual performance should be mentioned for describing this result. Previous studies showed that the reduction of visual performance in working people was related to the effect of illumination on the eye fatigue and mental fatigue [28, 29]. Shortage and deprivation in sleep are closely related to the reduction of visual performance. As mentioned in numerous studies, long shifts can interfere with the sleep-awakening cycle and reduce the quantity and quality of sleep [30]. One of the main disadvantages of 12-h shift that was mentioned in previous studies is sleepiness which can reduce the visual and vigilance performance and, on the other hand, increase the risk of accidents, and it has been proved that inappropriate illumination increases the level of sleepiness and eye fatigue [31]. It is in line with the study of S.D. Baulk et al., indicating that sleepiness increases significantly at the end of 12-h shifts [32]. This increase is certainly accompanied by a reduction in visual performances.

Therefore, it can be useful to use a lighting system with intensity of 400 lx and more, because of increased visual performance, reduction of eye fatigue and its complications such as headache, eye pain, dislike of work and etc. In the current study, the effect of the age and gender properties on the visual performance were not been examined. Therefore, it is recommended that these two factors be evaluated in the future studies.

## Conclusion

In general, it was discovered that the lighting system with intensity of 200 lx results in the reduction of the visual performance. And, the lighting system with intensity of 400 Lux can significantly improve visual performance. The results of the present study showed that increasing illumination from the values which are below the permitted level -for administrative work- to a higher level would increase the visual performance in individuals. Also, the results showed that time has a significant effect on visual performance, and visual performance changes during time. Considering the importance of visual performance in control-rooms, it is suggested that illumination should be increased in them - at least the final hours of the shift- in order to prevent errors and unexpected accidents.

## Abbreviations

FrACT: The Freiburg Visual Acuity test
IESNA: The Illumination Engineers Society of North America
LED: Light Emission Diode
SD: Standard Deviation
